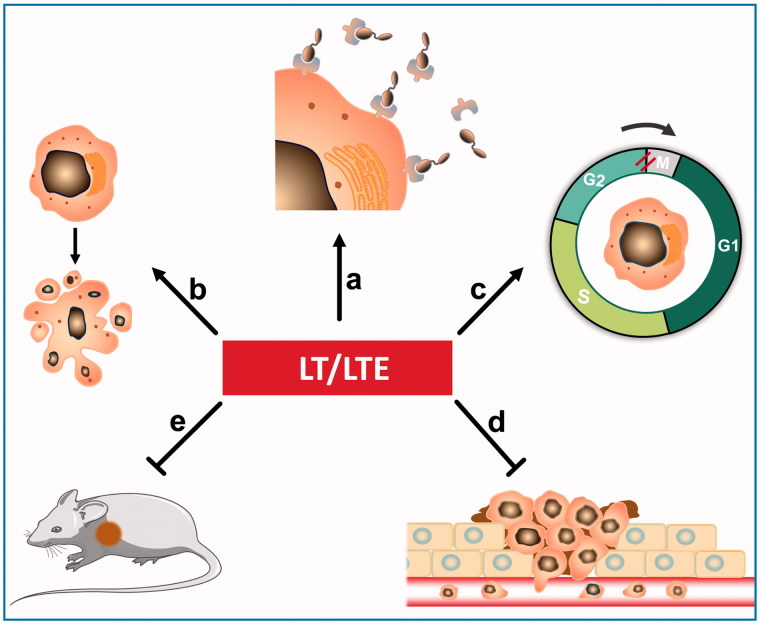# Intensive fibrosarcoma-binding capability of the reconstituted analog and its antitumor activity

**DOI:** 10.1080/10717544.2017.1410261

**Published:** 2017-12-17

**Authors:** Jian Xu, Yue Du, Wen-Juan Liu, Liang Li, Yi Li, Xiao-Fei Wang, Hong-Fei Yi, Chuan-Kun Shan, Gui-Min Xia, Xiu-Jun Liu, Yong-Su Zhen

**Affiliations:** ^a^ Institute of Medicinal Biotechnology, Chinese Academy of Medical Sciences and Peking Union Medical College Beijing China; ^b^ Shandong Provincial Key Laboratory of Radiation Oncology, Shandong Cancer Hospital and Institute, Shandong Cancer Hospital affiliated to Shandong University Shandong Academy of Medical Sciences Jinan China; ^c^ West China Hospital, Sichuan University and Collaborative Innovation Center Chengdu China

**Keywords:** Drug delivery, binding capability, treatment efficacy, evaluation, fibrosarcoma

## Abstract

Fibrosarcomas are highly aggressive malignant tumors. It is urgently needed to explore targeted drugs and modalities for more effective therapy. Matrix metalloproteinases (MMPs) play important roles in tumor progression and metastasis, while several MMPs are highly expressed in fibrosarcomas. In addition, tissue inhibitor of metalloproteinase 2 (TIMP2) displays specific interaction with MMPs. Therefore, TIMP2 may play an active role in the development of fibrosarcoma-targeting agents. In the current study, a TIMP2-based recombinant protein LT and its enediyne-integrated analog LTE were prepared; furthermore, the fibrosarcoma-binding intensity and antitumor activity were investigated. As shown, intense and selective binding capability of the protein LT to human fibrosarcoma specimens was confirmed by tissue microarray. Moreover, LTE, the enediyne-integrated analog of LT, exerted highly potent cytotoxicity to fibrosarcoma HT1080 cells, induced apoptosis, and caused G2/M arrest. LTE at 0.1 nM markedly suppressed the migration and invasion of HT1080 cells. LTE at tolerated dose of 0.6 mg/kg inhibited the tumor growth of fibrosarcoma xenograft in athymic mice. The study provides evidence that the TIMP2-based reconstituted analog LTE may be useful as a targeted drug for fibrosarcome therapy.

## Introduction

Cancer has been recognized as one of the major reasons for the increasing mortality (Hong et al., 2016). Among human malignancies, although fibrosarcomas are the relatively rare soft tissue tumors originating from intra- and intermuscular fibrous tissues, fascia, and tendons, they often display highly aggressive behaviors (Harati et al., 2016). Due to their lower frequency of occurrence, fibrosarcomas have not been studied intensively as the other malignant tumors (Min et al., 2017). Currently, for those cases of not suitable for surgical resection, fibrosarcoms are generally considered to treat with standard chemotherapy, radiotherapy as well as the other combinational regimens, however, the use of targeted drugs is limited (Riggi et al., 2007). Therefore, it is urgently needed to explore targeted drugs and modalities for more effective therapy.

Tissue inhibitor of metalloproteinase 2 (TIMP2), as a secreted endogenous protein by tumor cells and/or their associated stroma, attracted considerable attention for its participation in diverse tumorigenic processes (Moore & Crocker, 2012, Xia & Wu, 2015, Jackson et al., 2017). Particularly, accumulating studies have demonstrated that multiple matrix metalloproteinases (MMPs) endowed with the ability to degrade components of the extracellular matrix (ECM) are also overexpressed in fibrosarcomas, exerting complex effects on tumor progression, invasion, metastasis and angiogenesis (Maquoi et al., 1998, Togawa et al., 1999, Atkinson et al., 2004, Jablonska-Trypuc et al., 2016, Isaacson et al., 2017). Therefore, a comprehensive understanding of the TIMP2-MMPs-substrate axis is crucial in directing potential mechanism-based therapeutic interventions against fibrosarcomas.

As our previous study reported, the TIMP2-based recombinant protein LDP-TIMP2 (LT) and its enediyne-integrated analog LDP(AE)-TIMP2 (LTE) that targets MMP-14/MMP-2 were effective against human esophageal carcinoma xenograft model in nude mice (Xu et al., 2015). Considering the fact that several members of the MMP family are highly expressed in fibrosarcoma cells and TIMP2 may show specific interaction with MMPs, it is important to explore antitumor activity of the protein LT and its reconstituted analog LTE against fibrosarcoma. In the present study, intense and selective binding capability to human fibrosarcoma specimens was confirmed by tissue microarray, indicating the fibrosarcoma-oriented profile of the TIMP2-based protein. In addition, reconstituted analog LTE displayed highly potent cytotoxicity to fibrosarcoma HT1080 cells and inhibited the tumor growth of fibrosarcoma xenograft in athymic mice. Concurrently, the mechanism of action was investigated.

## Materials and methods

### Cell culture

The human fibrosarcoma HT1080 and lung carcinoma A549 cell lines were obtained from ATCC and maintained in modified RPMI-1640 (Hyclone, Logan, UT; Thermo Fisher Scientific, Waltham, MA) supplemented with 10% (v/v) of fetal bovine serum (Gibco, Carlsbad, CA; Life Technologies, Camarillo, CA) and 1% penicillin/streptomycin (North China Pharmaceutical, Hebei, China). Cells were cultured at 37 °C in a humidified atmosphere of 5% CO_2_.

### Preparation and characterization of the protein and active analog

*Pichia pastoris* strain GS115-pHBM-LDP-TIMP2 producing fusion protein LDP-TIMP2 (LT) was cultured in BMGY and BMMY medium, sequentially. After fermentation, the protein was purified by His Trap affinity columns (GE Healthcare, San Diego, CA). Briefly, the enediyne-integrated analog LDP(AE)-TIMP2 (LTE) was generated by reconstituting LDP and the active enediyne (AE) *in vitro* according to the previous procedure. The active analog LTE was determined by the reverse-phase HPLC system, using a Vydac C4 300A column (Grace, Williamsburg, MI). In addition, the molecular weight, far ultraviolet circular dichroism (CD) spectra, and isoelectric point (pI) of protein LT were detected by Sangon Biotech (Shanghai, China).

### *In vitro* binding analyses

*In vitro* binding activity of the FITC-conjugated ‘scaffold’ protein LDP and the delivery protein LT to HT1080 and A549 cells were detected by Flow Cytometry (BD FACS Calibur, San Jose, CA), and the binding intensity of serial concentrations of FITC-conjugated LT with the two cell lines was also determined. All assays were performed in triplicate. In addition, HT1080 cells were plated onto glass coverslips for further binding analysis using laser scanning confocal microscope (LEICA TCS SP5, Leica Microsystems GmbH, Wetzlar, Germany). All the above methods refer to the previous reports.

### Cell growth inhibition assay

The human cancer cells were plated at 3000 cells/well in 180 µL of growth medium in 96-well plates, and cultured at 37 °C overnight in a humidified CO_2_ incubator. Following, different tested agents including 20 µL of protein LT, active analog LTE were added into the pre-seeded wells for another 48 h incubation, respectively. After removal of supernatant, 100 µL of fresh medium and additional 10 µL of Cell Counting Kit-8 reagent (CCK-8) were added to each well of the plate successively. The absorbance was determined at 450 nm by a microplate reader (Thermo Fisher Scientific, Waltham, MA). Untreated cells served as the control. The optical density value (OD) represented the relative cell viability.

### Analysis of cell-cycle arrest

Analysis of cell-cycle arrest was conducted by Flow Cytometry (BD FACS Calibur, San Jose, CA) (Xu et al., 2017). Briefly, after the growth reaching 50% confluence in 6-well plates, HT1080 and A549 cells were treated with different concentrations of LTE for 24 h. Following, cells were collected and washed with cold PBS, then fixed with 5 mL of 70% ethanol at −20 °C overnight. The cells were washed, and re-suspended with fresh PBS, containing 50 µg/mL propidium iodide (PI) and 100 µg/mL RNAse A. After 30 min incubation at RT, cells were analyzed for DNA content. All experiments were independently carried out for three times.

### Determination of apoptosis using Annexin V/PI staining

Detection of apoptosis was conducted by Annexin V-FITC Apoptosis Assay Kit (Biosea Technology, Beijing, China) according to the manufacturer’s protocol. Briefly, the HT1080 and A549 cells were cultured in 6-well plates and treated with different concentrations of the active analog LTE for 24 h incubation. Then, cells were harvested and washed with cold PBS. Following, cells were re-suspended in the binding buffer. The cells were then incubated with Annexin V-FITC/PI and maintained in the dark room. Analysis was performed using Flow Cytometry (BD FACS Calibur, San Jose, CA). All experiments were independently repeated three times.

### Migration and invasion assays

The migration of HT1080 cells was determined by wound-healing assay (Yu & Kim, 2016). Briefly, HT1080 cells were seeded into 6-well plates. After reaching 85% confluence, a scratch was made among cells with a sterile 20 µL of pipette tip. The floating cells were removed by PBS washing, and fresh medium (or with a lower concentration of FBS) containing different tested agents was added. The cells were further cultured for 24–48 h and observed under an inverted microscope (TE2000-U, Nikon, Tokyo, Japan). The representative images were photographed and distances between scratch edges were measured and statistically analyzed.

The invasion of HT1080 cells was determined with a 24-well, 8.0 µm pore size Trans-well plate (Corning Costar, Corning, NY) (Yu & Kim, 2016). A 6.5 mm diameter insert membrane was coated with 100 µL of BD Matrigel^TM^ Basement Membrane Matrix (0.5 mg/mL in ice-cold RPMI-1640) and then dried at 37 °C for 2 h. The matrigel coated plates were rehydrated in 200 μL serum-free RPMI-1640 medium and incubated at 37 °C for 30 min before the experiment. After removing the rehydration medium from the insert, 600 µL of fresh medium containing 20% FBS was added to the lower chamber, 100 µL of cell suspension (1 × 10^5^ cells/well) in serum free medium (containing different concentrations of the active analog LTE) was plated in the upper chamber. After incubation at 37 °C for 24 h, the inserts were fixed with methanol at RT for 30 min and followed by 0.1% (w/v) crystal violet staining at RT for another 30 min. Cotton swabs were used to remove non-invade cells from the upper chamber. Following, the representative images were photographed with an inverted microscope (TE2000-U, Nikon, Tokyo, Japan). For further objective evaluation, the cropped membrane was incised and dipped into 150 µL of 33% acetic acid to dissolve crystal violet at RT for 10 min on a horizontal shaker. Finally, the solution was transferred to 96-well plates, respectively. The absorbance was recorded at 570 nm with a microplate reader (Thermo Fisher Scientific, Waltham, MA). Each experiment was performed for three times independently.

### Human tumor specimens and immunohistochemical staining

Human paraffin embedded tissue array (soft tissue, SO2084, 104 cases/208 points) was ordered from AlenaBio (Xi’an, China). Following, the detection and analysis were performed by Shanghai Outdo Biotech Co., Ltd. (Shanghai, China). The protein LT was used as the primary binding agent with tumor cells.

### Mouse xenograft model and treatment

Studies involving the use of animals were approved by the Ethics Committee for Animal Experiments of the Institute of Medicinal Biotechnology, Chinese Academy of Medical Sciences (IMBF20060302). The experimental procedures were conducted with strict adherence to the National Guidelines for Housing and Care of Laboratory Animals.

Female athymic mice (BALB/c, *nu/nu*, 18–22 g) were purchased from Vital River Laboratories (Beijing, China). Fibrosarcoma HT1080 cell suspensions (1 × 10^7^ cells in 200 µL of physiological saline) were implanted subcutaneously (s.c.) into the right armpit of 8-week-old mice to establish xenograft tumors. After 3 weeks, the tumors were dissected aseptically and pieces of the tumor tissue (2 mm^3^ in size) were transplanted into mice subcutaneously using a sterile trocar. When the tumor volume reached around 100 mm^3^, mice were randomized into five groups (six mice in each group) and intravenously injected with the protein LT, and three different dosages of LTE. Additionally, a group that received physiological saline served as the vehicle control. During the entire experiment, a total of two injections with a 7-d interval were performed. Meanwhile, body weight of mice was measured at the appointed days (Zheng et al., 2017). The experiment was ended at 30 d after tumor transplantation. Mice were humanely killed, and the xenografts were dissected and weighed. Finally, the fresh tumor tissue specimens were collected for histochemical study.

### Statistics

Data were presented as mean ± standard deviation (SD), and analyzed using GraphPad Prism 6.0 software (GraphPad Software, La Jolla, CA). Statistical significance established at *p* < .05.

## Results

### Preparation and characterization of protein LT and active analog LTE

**Figure 1.**
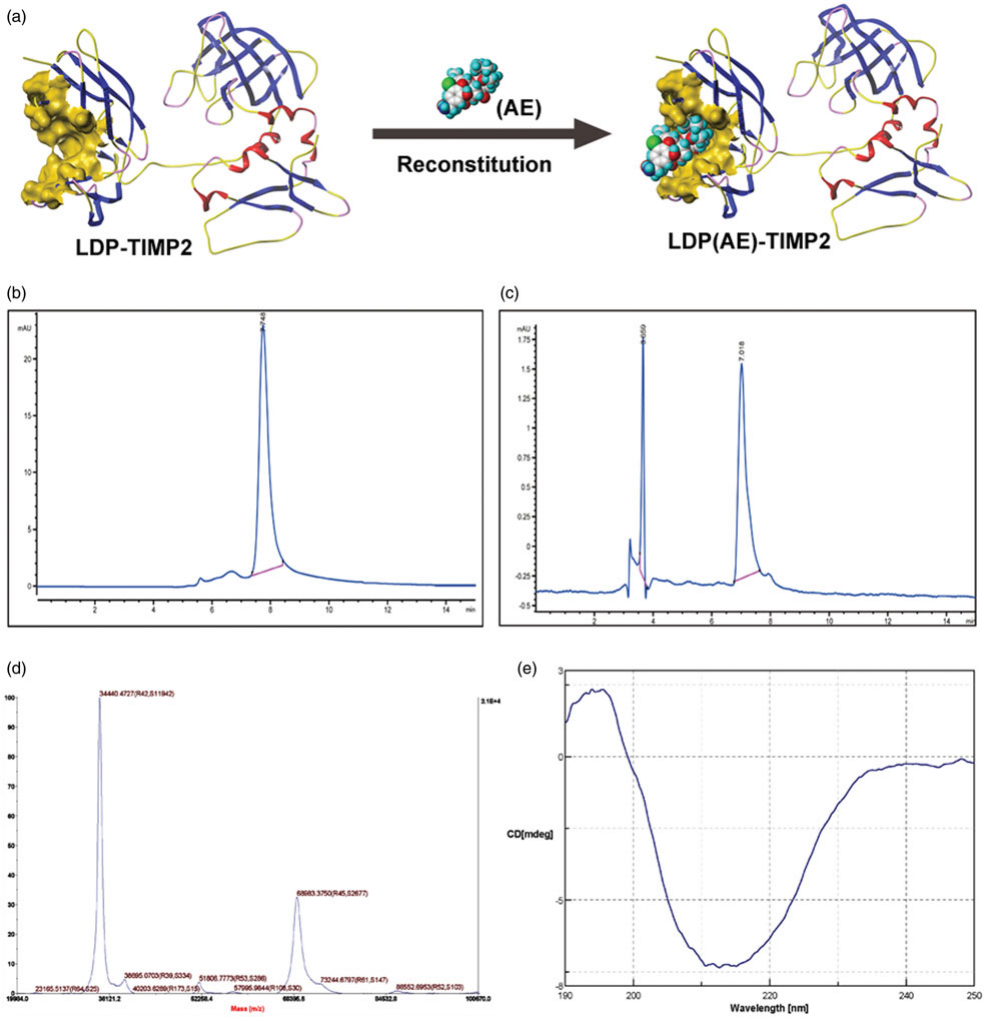
Preparation and characterization of the protein LT and its active analog LTE. (a) The schematic diagram for conformation of the recombinant protein LDP-TIMP2 (LT) and the reconstitution of its enediyne-integrated analog LDP(AE)-TIMP2 (LTE). (b) The protein purity analysis using HPLC system. (c) The reconstitution of enediyne-integrated analog LTE, determined by reverse-phase HPLC system. (d) Molecular weight of the protein LT, measured by 5800 MALDI-TOF/TOF system. (e) The far-UV (190–250 nm) CD spectra of the protein LT, analyzed by JASCO715 system.

According to amino acids composition, the conformation of protein LT and enediyne integrated analog LTE, as well as *in vitro* reconstitution is shown in Figure 1(a). Because the 6 × his tag was added at the C-terminus of the protein, His Trap Affinity Columns were used for purification. The purity of protein LT was detected by HPLC as shown in Figure 1(b). The assembly efficiency was determined by reverse-phase HPLC as shown in Figure 1(c). The molecular weight of protein LT was 34.4 kD as determined by 5800 MALDI-TOF/TOF system (Figure 1(d)), which was consistent with its theoretical value of amino acid composition; in addition, a potential dimer with a molecular weight of 68.9 kD was also detected. The result of far ultraviolet circular dichroism (CD) spectra (Figure 1(e)) showed that the α-helix was about 3.0%, the β-pleated sheet 76.3%, the turn 0.0%, and the random coil 20.8%, which also demonstrated the secondary structure of the protein LT. By using GE Healthcare multiphor II electrophoresis system, the isoelectric point (pI) of the protein was 6.01.

### *In vitro* binding capability of protein LT

**Figure 2.**
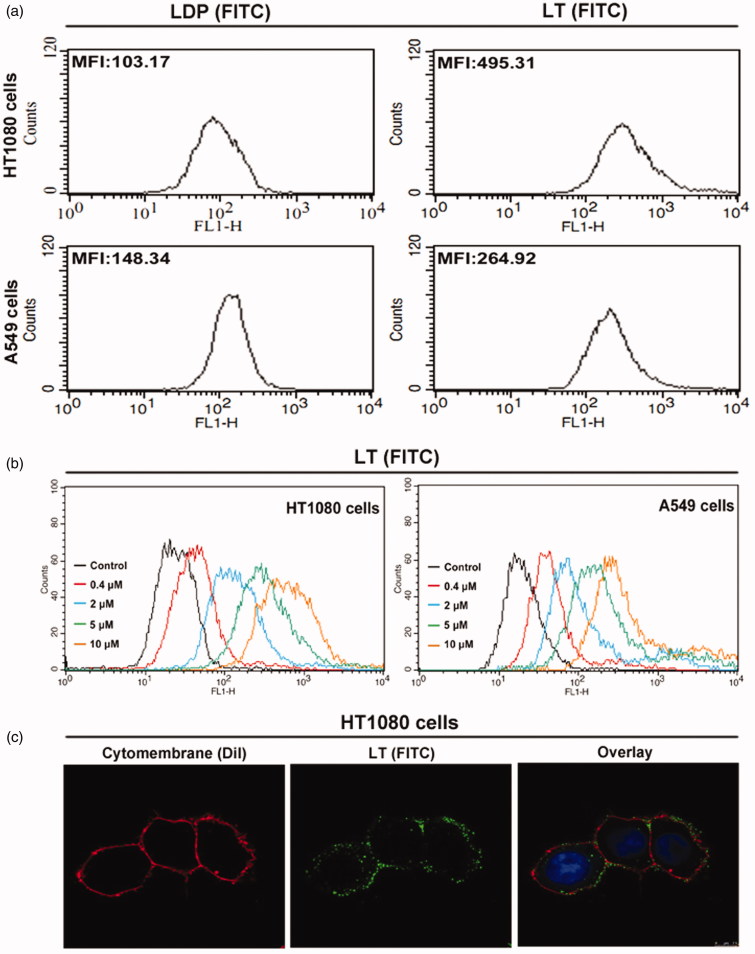
*In vitro* binding activity analyses. (a) Binding efficiency of 5 μM FITC-conjugated protein LT or LDP to HT1080 and A549 cells, determined by FACS analysis. The experiment was carried out in triplicate. (b) Binding activity of different concentrations of FITC-conjugated protein LT to HT1080 and A549 cells via FACS analysis. HT1080 or A549 cells without LT (FITC) served as the control. Each experiment was performed in triplicate. (c) Binding affinity of the protein LT to living HT1080 cells was analyzed by confocal microscope, the representative image was obtained by the LEICA TCS SP5 System (Leica Microsystems GmbH, Wetzlar, Germany). The overlay image was with FITC-conjugated protein LT (green), DiI staining (red), and DAPI staining (blue).

In a previous study, moderate level of MMP-2 expression was confirmed in both HT1080 and A549 cells, while only high level of MMP-14 expression was detected in HT1080 cells. Additionally, the Co-ip analysis was also employed to verify the binding capability of protein LT to MMP-14 and MMP-2 concurrently in HT1080 cells (data not shown). Herein, the FITC-conjugated protein LT and LDP were used to further demonstrate the binding ability via FACS analysis. As the MFI value (mean fluorescence intensity) presented in Figure 2(a), the binding capability of LT (FITC) to both HT1080 and A549 cells was stronger than that of LDP (FITC). Moreover, different concentrations of protein LT (FITC), ranging from 0 to 10 µM, were incubated with cells at RT for 2 h, and MFI was also detected and analyzed via FACS system. The results (Figure 2(b)) showed that the binding efficiency of protein LT to HT1080 cells was stronger than to A549 cells via potential MMP-14/MMP-2 double-directing. In addition, for further exploring the binding capability with living HT1080 cells, the confocal-based detection was performed. After 2 h incubation at 37 °C and sample processing, the green fluorescent signal distribution in cytomembrane was detected (Figure 2(c)), suggesting the highly binding capability of protein LT (FITC). Results were obtained from three times of repeated experiments.

### Binding capability analysis using tissue microarray

**Figure 3.**
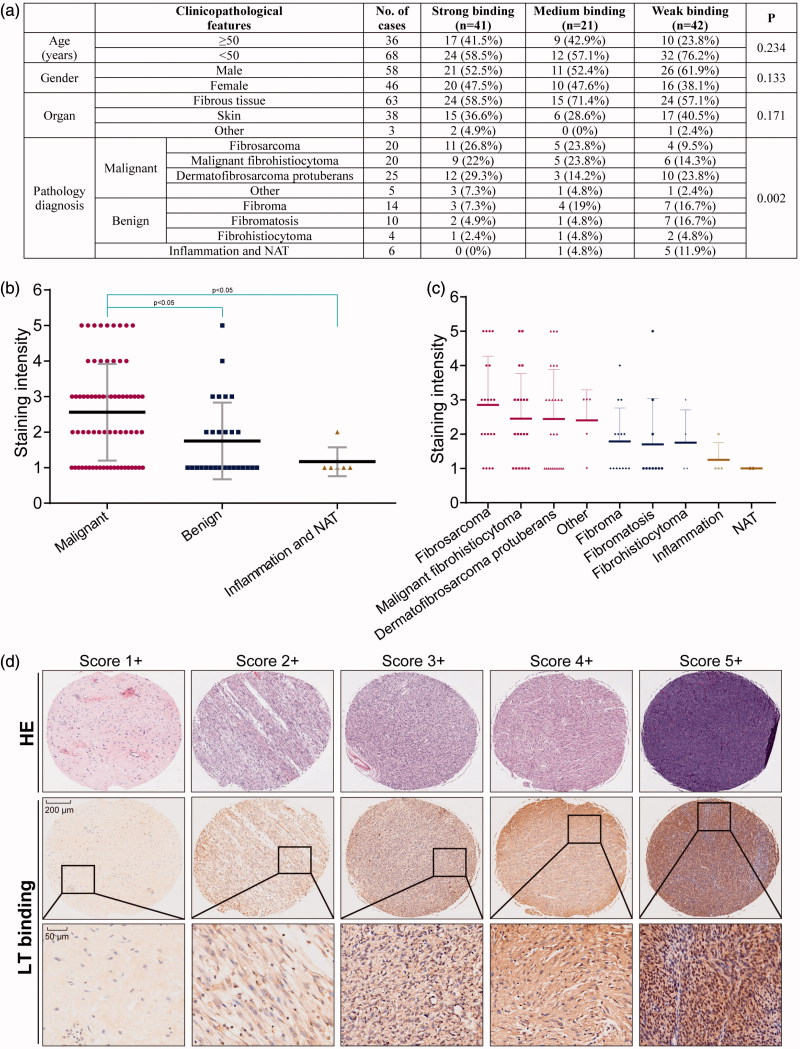
Binding capability of the protein LT via tissue microarray. (a) Different classifications according to the clinicopathological features, such as age, gender, organ, and pathology diagnosis. Score 1 + indicates the weak binding, score 2 + indicates the moderate binding, and score 3 + or more indicates strong binding. Student’s *t*-test was used for analyzing the statistical difference among groups. Data were analyzed by one-way ANOVA in the table. (b) The classification of staining intensity in malignant, benign and inflammation (and NAT), *p < .05, showing significant difference. (c) The classification of staining intensity according to pathology diagnosis. (d) Representative cases showing classification of binding capability. The HE staining was performed by AlenaBio (Xi’an, China), and used for showing tumor area.

The final score demonstrating binding capability was obtained by multiplying the intensity and extension values, and the samples were grouped as 1+, 2+, 3+, 4+, 5+. Meanwhile, for statistical purpose, the score of 3+, 4+ , and 5 + were defined as strong binding, 2 + was defined as moderate binding, and 1 + or others were considered as weak binding. As the results shown, much higher percentage of cases of the fibrosarcoma and related malignancies displayed strong and moderate binding, indicating the targeting capability of the recombinant protein LT to soft tissue malignant tumors (Figure 3). 

### Bioactivity analyses of protein LT and the enediyne-integrated analog LTE

**Figure 4.**
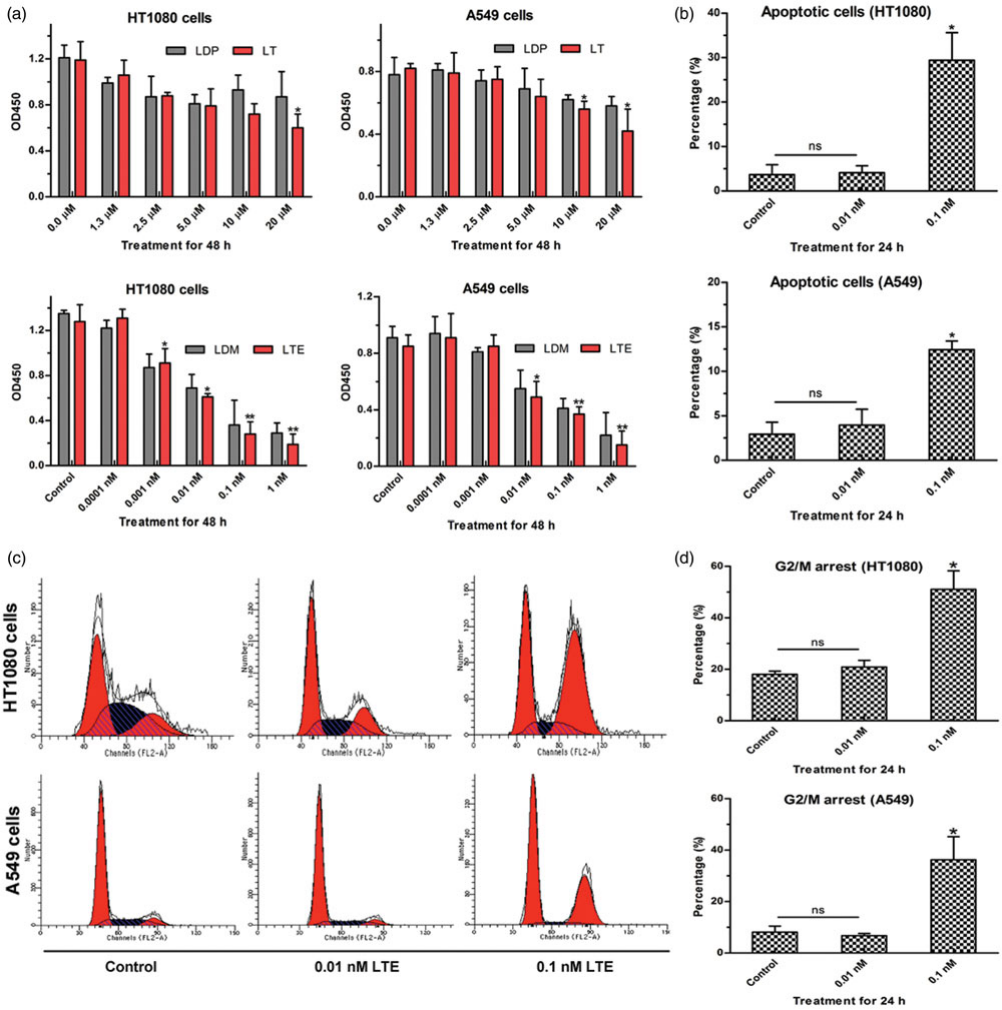
The bioactivity analyses of protein LT and active analog LTE. (a) HT1080 cells treated with protein LT and LDP for 48 h (upper left), A549 cells treated with protein LT and LDP for 48 h (upper right); HT1080 cells treated with the active analog LTE and LDM for 48 h (lower left), A549 cells treated with the active analog LTE and LDM for 48 h (lower right). *p < .05, versus control, without treatment with tested agent, while **p < .01, versus control. The experiment was performed using Cell Counting Kit-8 assay. Results were obtained from six times of repeated experiments. (b) The proportion of apoptotic cells (the early and late) was presented as the average value of three times of independent experiments, respectively. (c) Active analog LTE-induced G2/M arrest in HT1080 and A549 cells after 24 h incubation. (d) Percentage of G2/M arrest in HT1080 and A549 cells treated with different concentrations of the active analog LTE. Statistical analysis of three times of independent experiments. Cells without treatment served as the control. *p < .05, against control. Each experiment was performed in triplicate.

To investigate the cytotoxicity of protein LT (LDP, served as the control), and enediyne-integrated analog LTE (LDM, served as the control) to HT1080 and A549 cells, Cell Counting Kit-8 assay was performed. Different concentrations of protein LT and LDP (0 µM, 1.3 µM, 2.5 µM, 5 µM, 10 µM, and 20 µM) as well as the LTE and LDM (0 nM, 0.0001 nM, 0.001 nM, 0.01 nM, 0.1 nM, and 1 nM) were employed for analyses. As shown in Figure 4(a), when treated with 20 µM of protein LT, both the growth of HT1080 (upper left) and A549 cells (upper right) were inhibited moderately, and significant difference was shown as compared with the control (0 µM), respectively. Additionally, in Figure 4(a) (lower left and right), more striking inhibitory potency was confirmed, which also demonstrated the successful reconstitution of active enediyne to the protein LT.

For analysis of the bioactivity of active analog LTE, FACS-based Annexin V-FITC/PI Apoptosis assay was performed. As presented in Figure 4(b), when treated with 0.1 nM LTE for 24 h incubation, the proportion of apoptotic cells increased to 29.43% in HT1080 cells, while 12.45% in A549 cells. Evidently, HT1080 cells were more sensitive to the tested LTE. The data represented the average value of three independent experiments.

For further determination of the G2/M arrest of HT1080 and A549 cells induced by the active analog LTE, FACS-based DNA content analyses were carried out. As shown in Figure 4(c), when treated with LTE for 24 h incubation, G2/M arrest of HT1080 and A549 cells increased significantly in a concentration-dependent manner, while Figure 4(d) suggests three independent experiments.

### Invasion and migration assays

**Figure 5.**
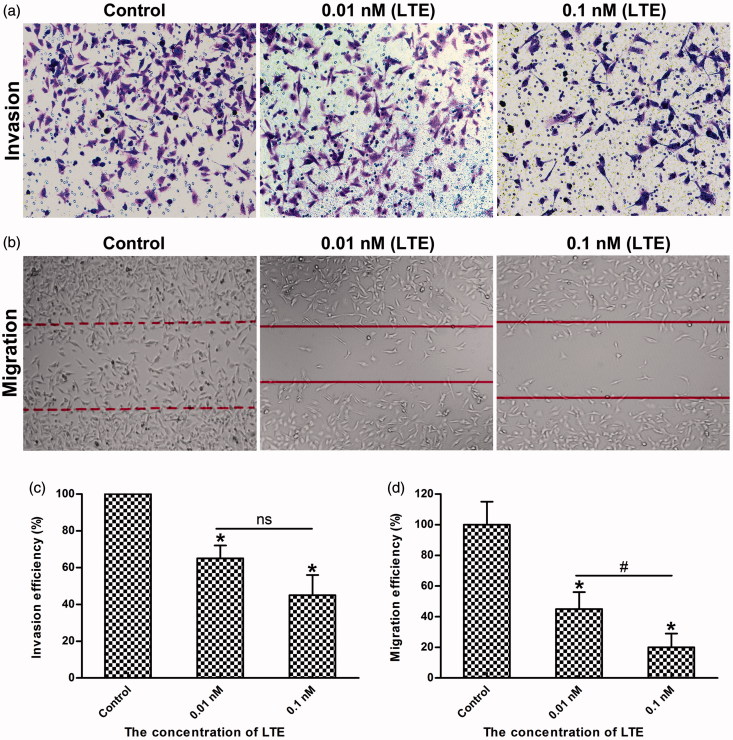
LTE inhibited the invasion and migration of HT1080 cells. (a) The representative images of LTE-induced inhibition of invasion in HT1080 cells after 24 h incubation. (b) The representative images of LTE-induced inhibition of migration in HT1080 cells after 24 h incubation. (c) Statistical analysis shows that LTE treatment caused a significant decrease in cell invasion. (d) Statistical analysis shows that LTE treatment caused a significant decrease in cell migration. In both invasion and migration analyses, cells without treatment served as the control. Columns, mean; Error bars, SD. *p < .05, against control. ^#^ *p* < .05, against 0.01 nM treatment group. Each experiment was performed in triplicate.

Tumor cells invasion and migration are important characteristics not only for cancer development but also for cancer metastasis (Yasue et al., 2010, Steeg, 2016). As reported, MMP-2 and MMP-14 (MT1-MMP) are usually overexpressed in tumor cells, which play important roles in promoting cancer cell invasion and migration. To verify the inhibitory effects of LTE on HT1080 cells migration and invasion, wound-healing assay and matrigel-based transwell assay were used for evaluation. As shown in Figure 5(a), when treated with 0.01 nM and 0.1 nM active analog LTE for 24 h incubation, the invasion of HT1080 cells was inhibited evidently, as compared with the control (*p* < .05), while Figure 5(c) shows statistical analysis of three independent experiments. In addition, as presented in Figure 5(b), when treated with 0.01 nM and 0.1 nM of LTE for 24 h incubation, the migration of HT1080 cells was also suppressed obviously, as compared with the control (*p* < .05), while Figure 5(d) also shows statistical analysis of repeated experiments, significant difference was found between the 0.01 nM and the 0.1 nM treatment group.

### *In vivo* therapeutic efficacy of active analog LTE

**Figure 6.**
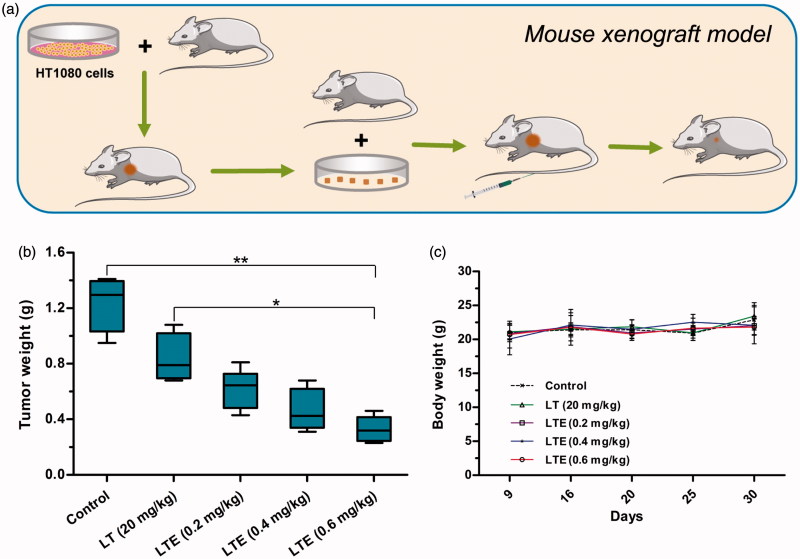
*In vivo* antitumor efficacy of LTE against fibrosarcoma HT1080 xenografts in athymic mice. (a) Mouse xenograft model and treatment. (b) Tumor weights in different treatment groups and the control group. *p < .05, the 0.6 mg/kg LTE group versus 20 mg/kg LT group; **p < 0.01, the 0.6 mg/kg LTE group versus the control group. (c) Body weight change of the HT1080 xenograft-bearing mice during the observation period.

The antitumor efficacy of the active analog LTE (0.2 mg/kg, 0.4 mg/kg, and 0.6 mg/kg) and protein LT (20 mg/kg) was investigated using fibrosarcoma HT1080 xenografts in athymic mice (Figure 6(a)). Treatment agents and saline control were administrated to tumor-bearing mice intravenously at the day 9 and day 16 after subcutaneous tumor transplantation. At the termination of the experiment, tumors were removed from the mice and weighed. Tumor weights were presented, suggesting that the 0.6 mg/kg LTE obviously suppressed the growth of HT1080 xenografts, as compared with the saline control group (Figure 6(b)). No animal death was found in the control and treatment groups. There was no significant change in body weight in treatment groups during the course of observation (Figure 6(c)), indicating that the doses of the tested agents were well tolerated.

## Discussion

Fibrosarcomas are low incidence of soft-tissue sarcomas and generally less sensitive to chemotherapy and radiotherapy in clinical practice. Overall, the standard treatment regimen comprises surgical resection, and then in terms of the recovery situation, followed by some other adjuvant treatments to reduce the local recurrence rate (Karavasilis et al., 2008, Patrikidou et al., 2011, Gronchi & Casali, 2013). Despite the local control is effectively improved, distant metastasis still occurs in nearly 50% of all patients, showing poor results and no significant improvements in overall survival (Harati et al., 2016). Currently, the chemotherapeutic agents, doxorubicin and ifosfamide, are most frequently used in soft tissue sarcomas treatment, however, only 20–30% response rate was achieved in the disseminated disease, so the combination with other agents is also a promising strategy (Bui-Nguyen et al., 2012, Radaelli et al., 2014, Gordon et al., 2016). It is urgently needed to develop rational treatments, particularly for metastatic or unresectable fibrosarcomas.

In previous study, we prepared a TIMP2-based recombinant protein and its enediyne-integrated analog, and subsequently, demonstrated their antitumor efficacy against human esophageal carcinoma xenografts in athymice mice. The TIMP2-based protein displayed selective binding capacity to the tumor tissues via the interaction with MMP-14/MMP-2. Considering the fact that members of the MMP family might play a critical role in tumor progression and metastasis, TIMP2 was further investigated for the potential efficacy on fibrosarcoma and related soft tissue malignancies (Nonaka et al., 2005). In the present study, the intense binding capability of protein LT to fibrosarcoma and related soft tissue malignancies was detected by tissue microarray. In addition, LTE, the enediyne-integrated analog of LT, displayed highly potent cytotoxicity to fibrosarcoma HT1080 cells and lung carcinoma A549 cells; in comparison, the former was more sensitive to LTE than the latter. The present study found that LTE markedly suppressed the growth of fibrosarcoma HT1080 xenografts in athymic mice. We found that the antitumor effect of LTE may be ascribed to cell proliferation inhibition and apoptosis induction. As shown, LTE also induced apoptosis and caused G2/M cell-cycle arrest in HT1080 and A549 cells, suggesting that the intracellular signaling pathway of apoptosis was affected by LTE. Moreover, LTE strongly inhibited the invasion and migration of HT1080 cells, which were mainly involved in tumor metastasis. With a highly potent invasive potential, the fibrosarcoma HT1080 cells exhibit aberrant secretion of MMP-2 and MMP-14, which acts as the receptor of TIMP2; accordingly, this may be the mechanistic basis for selective intense binding of the TIMP2-based protein LT to fibrosarcomas. In this case, TIMP2-based protein LT actually served as the fibrosarcoma-oriented delivery carrier, and the integrated enediyne molecule acted as the highly potent effector agent.

## Conclusions

Taken together, these data provide evidence that the TIMP2-based recombinant protein LT endowed with binding capability to fibrosarcoma cells could serve as the carrier of targeting delivery, in addition, the enediyne-integrated analog LTE could act as a targeted agent. Further evaluation of the antitumor efficacy and elucidation of the target-agent interaction are required.